# The Effect of Weekly Training Load across a Competitive Microcycle on Contextual Variables in Professional Soccer

**DOI:** 10.3390/ijerph18105091

**Published:** 2021-05-11

**Authors:** Marcos Chena, José Alfonso Morcillo, María Luisa Rodríguez-Hernández, Juan Carlos Zapardiel, Adam Owen, Demetrio Lozano

**Affiliations:** 1 Faculty of Medicine and Health Sciences, Campus Universitario-C/19, University of Alcalá, Av. de Madrid, Km 33,600, Alcalá de Henares, 28871 Madrid, Spain; marcoschenapf@hotmail.com (M.C.); mluisa.rodriguez@uah.es (M.L.R.-H.); carlos.zapardiel@uah.es (J.C.Z.); 2 Departamento de Didáctica de la Expresión Musical, Plástica y Corporal, University of Jaén, s/n, 23071 Jaén, Spain; jamlosa@ujaen.es; 3 Centre de Recherche et d’Innovation sur le Sport (CRIS), Lyon University, 92 Rue Pasteur, 69007 Lyon, France; adamowen@outlook.com; 4 Health Sciences Faculty, Universidad San Jorge, Autov A23 km 299, Villanueva de Gállego, 50830 Zaragoza, Spain

**Keywords:** football, GPS, performance, external load, match load, contextual factors

## Abstract

Analysis of the key performance variables in soccer is one of the most continuous and attractive research topics. Using global positioning devices (GPS), the primary aim of this study was to highlight the physiological response of a professional soccer team across competitive microcycles in-season according to the most influential contextual performance variables. Determining the training load (TL), a work ratio was established between all recorded data within the training sessions and the competitive profile (CP). Each microcycle was classified in accordance with the contextual variables: opponent level (high, medium, low), match location (home and away) and score (win, draw, lose). Results revealed that the team were significantly more successful (games won) in competitive games against high-level opponents and when played at home. Cumulative microcycle/weekly training load (WTL) was significantly lower when the team won. In addition to the opponent level and the match location, WTL could condition the athlete’s performance in the competition. Competitive performance responses are the main source of information for the planning of training programs. The results of this study could be used as a reference to structure TL and WTL according to contextual variables in the competition. This study, which is the first of its kind, revealed that WTL effects the performance of the players in the competition.

## 1. Introduction

Analysis of the performance variables in team sports has been one of the most noted research topics within professional soccer in recent years [1,2,3,4,5,6]. The physical, technical and tactical demands of professional soccer players have continued to evolve, according to recent research [1,4], with one of the main sources of information concerning the planning of training programs [1,6]. From recent findings in the literature, the work-rate profile may be altered due to a large number of factors, including the methods used to determine soccer players movement profiles or time motion analysis [7].

Training load (TL) monitoring and the assessment of elite players in professional soccer through the use of global positioning systems (GPS) is well documented and performed on a daily basis [1,5,8]. GPS have become increasingly frequent to analyze the competitive performance variables in professional soccer teams [1,5,9]. Although it is a tool to quantify the external load, strong correlations have been found with other methods of quantification of internal load based on session-RPE [10,11].

Over the decades, there has been a need to control technical and physical elements in an isolated way to predict the team’s performance in competition [2,3,9,12,13]. However, current research trends have shown that success in soccer is multifactorial and complex [8]. Physical and technical responses are determined by the tactical behaviors in the competition [1]. Contextual variables condition competitive demands [6,14,15,16,17]. The most influential contextual variables in performance were opponent level (high, medium, low), match location (home and away) and score (win, draw, lose) [6,14,17].

The training sessions are grouped following different logical criteria to organize the planning of the physical elements, among others, of the season. One of the minimum groupings or units used in the professional football literature studied is the competitive microcycle [18,19]. The competitive microcycle is the one comprised of a block of training sessions carried out during a week included in the competition period and that includes at least one official match [18]. This type of structured microcycles contains the stimulation or loading phase, the tapering phase, and the competition phase [8,20].

Recent literature has reported that enabling variation among specific training parameters may maximize training adaptations while minimizing the accumulative effects of fatigue [10,21,22]. Although competitive physical responses in professional soccer players have been described with precision according to contextual variables in scientific literature [6,14,16,17], no study has revealed the physical response of the professional soccer players during the training weeks according to these contextual variables. Therefore, the aim of this study was to describe the cumulative weekly training load (WTL) as physiological responses of a professional soccer team during the competitive microcycles attending to the most influential contextual variables in performance.

## 2. Materials and Methods

### 2.1. Participants

Twenty-two professional soccer players participated in this study. The average age, height, weight, % body fat, VO2max (Yo-Yo Intermittent Recovery Test level 2) and sum of six skinfold sites were 26.36 ± 4.0 years, 179 ± 5.8 cm, 74.76 ± 8.0 kg, 9.88 ± 8.0%, 54.3 ± 5.1 mL·kg^−1^ and 39.67 ± 13.6 mm, respectively. Informed consent was obtained from all players to include their data after a detailed explanation about the investigation. Players were told that they were free to withdraw their information and data from the study at any time and the study conducted was fully approved by the involved Sports Management Department at the Football Club. Furthermore, the study met the ethical standards of research from the University of Alcalá, Madrid, Spain, with the code CEI/HU/2019/08, and was conducted in accordance with the principles outlined in the Declaration of Helsinki. Players were instructed to maintain normal daily food and water intake. All players had breakfast together every day and ate at least 4 days a week after the training under the supervision of the club. No additional dietary interventions were undertaken throughout the investigation.

### 2.2. Study Design

This is a retrospective observational research study conducted with professional soccer players during a season. Throughout the investigation, a total number of 30 competitive microcycles with 120 training session data was collected for assessment and analysis. According to Owen et al. [23], the data assessed included 30 × 1-week blocks of full training sessions per player, per position: 30 × match day 4 (MD-4), 30 × match day 3 (MD-3), 30 × match day 2 (MD-2) and 30 × match day 1 (MD-1).

Throughout the investigation, the data of 30 competitive microcycles with 120 training sessions were collected for assessment and analysis. According to Owen et al. [23], the data assessed included 30 microcycles per 1-week blocks of full training sessions per player, per position. Each weekly block is made up of the day of the weekly match (match day (MD)) and four training days called match day 4 (MD-4) (training performed 4 days before the match), match day 3 (MD-3), match day 2 (MD-2) and match day 1 (MD-1).

No other data pre-MD-4 was considered for analysis as they did not have any specific on pitch content of adequate TL. For the reliability and validity of the study, only data from players who performed the full session duration have been used, withdrawing the data from the goalkeepers and players whose TL was manipulated during this time due to fatigue management or injury. Players included within the data collection were only inclusive of players who had performed greater than 80 min of the competitive match-play at the end of the analyzed week [8]. All players were fully familiarized with the experimental procedures and the requirements of the training weeks assessed prior to the present study and were extremely familiar with the use of GPS units worn throughout the investigation.

### 2.3. Time Motion Analysis

Time motion of each player was recorded individually in all training sessions and matches through a 10 Hz GPS device (GPEXE ©, Udine, Italy) as used in previous studies [24,25]. Research has shown this system to be a valid and reliable assessment for monitoring team player’s movement demands [24]. To avoid variability, each player always uses the same GPS device, located between the two scapulae through a special vest. The specialized analysis software GPEXE © was used to download the data. For the purpose of this study, four volume metrics and four intensity metrics were recorded throughout the sessions, being a sufficient number of variables to provide relevant information about TL [8,25]. Volume metrics included: total distance covered (TDC), total number of high accelerations (ACC), total number of high decelerations (DEC) and high-speed running (HSR) as used in previous studies [8,23]. Intensity metrics included: equivalent distance index (EDI), metabolic power (MP), relative distance covered (RTDC) and relative high-speed running (RHSR) (Table 1).

### 2.4. Quantification and Management of TL

The methodology used to quantify TL according to the competition requirements consisted in to determine a ratio between the data recorded in the training session and the competitive profile (CP) (training session/CP) representation in %. In order to attain the daily volume and intensity scores, average individual player data were pooled to provide a squad average to create an initial session metric outcome score. The mean individual data assessed were then compared to the average maximum individual competitive match-play metric achieved (CP) and reported as a percentage figure as used in previous studies [8]. WTL was described as the cumulative weekly training load and was calculated with the sum the TL values of each training session and the competition load (WTL = MD-4 + MD-3 + MD-2 + MD-1 + MD) [26].

### 2.5. Contextual Variables

Three independent variables were included in the research. With respect to the contextual variable match location and in line with previous studies [6,15], we distinguished between matches played at home and away. With respect to the opponent level, we examined differences in physical performance when the reference team played against successful teams (ranked in the top 7 league positions = high), moderately successful teams (ranked from 8th to 16th in the league = medium) and the least successful teams (ranked in the bottom 7 of the league = low). These categories are similar to those reported previously [15]. With respect to the final result, this was divided into win, lose or draw.

### 2.6. Statistical Analyses

Data are presented as the mean ± standard deviation (±SD). Before using parametric tests, the assumption of normality was verified using Kolmogorov–Smirnov. The use of one-way analysis of variance for repeated measures to examine the difference in the GPS metrics collected between days. When significant *p* values were observed within the data (*p* < 0.05), *t*-test paired comparisons were used in conjunction with Holm’s Bonferroni method. Differences in time motion with respect to the 3 independent variables were determined using the Student’s *t*-test (match location) and a one-way analysis of variance (opponent level and score). When a significant F-value was found, Bonferroni’s post hoc tests were applied. The level of statistical significance was set at *p* < 0.05. Analyses were performed using SPSS for Mac version 24.0 (SPSS Inc., Chicago, IL, USA).

## 3. Results

The basic descriptors, 120 training sessions (30 × MD-4, 30 × MD-3, 30 × MD-2 and 30 × MD-1) and 30 matches (30 × MD) were analyzed (Table 2).

In order to attain the daily volume and intensity scores, average individual player data were pooled to provide a squad average to create an initial session metric outcome score. Metrics registered by the team in each training session were compared with the CP to determine % TL according to CP (Table 3).

**Figure 1:**
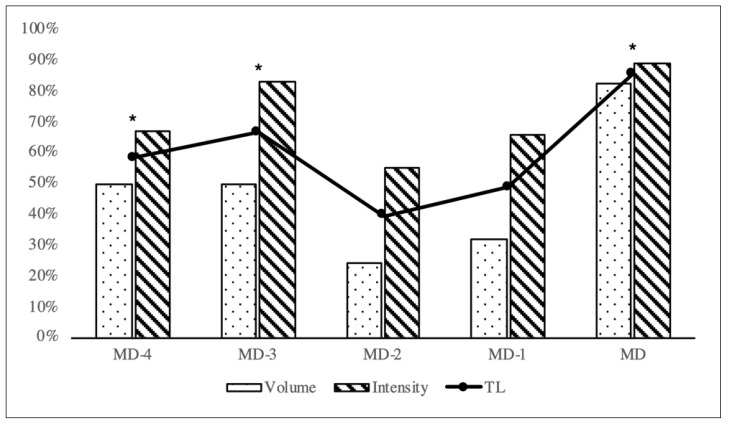
Representation of the average values of the volume, intensity, and training load (TL). MD 1–4: match day. * Significant differences found for session training load score between each training day across the microcycle (*p* < 0.05).

Metrics registered by the team in each training session were compared with the CP to determine % TL according to CP (Table 3). The volume and intensity fluctuated throughout the competitive week in relation to MD. After analyzing 30 competitive microcycles, it was observed that intensity was greater than volume in all training sessions. TL scores for MD, MD-4 and MD-3 were significantly higher than MD-1 and MD-2. However, no differences were found between MD-1 and MD-2 (Figure 1).

WTL had an average of 298.2% more load than CP. EDI, MP and RTDC were the metrics that increased as WTL accumulated (375%, 378.8% and 398% with respect to CP) (Table 3).

As seen in Table 4, WTL was significantly higher in the microcycles when the team played against the least successful teams (low level) compared to when the team played against high level opponents (306.7% vs. 292.1%). Decelerations accumulated during the microcycles (WTLDec) when the team lost were significantly greater than when the team drew.

**Figure 2:**
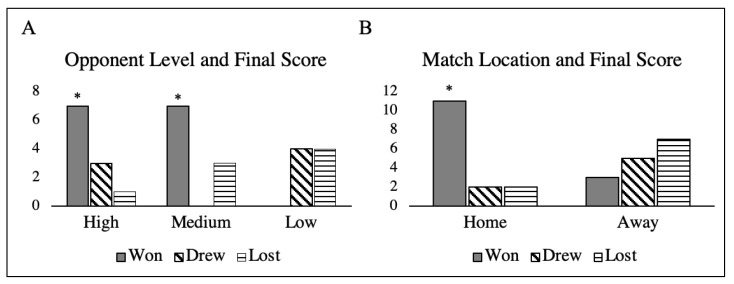
(**A**) Representation of final score of the team according to opposition’s level and (**B**) match location. * Significant differences between to win, draw and lose according to opponent level and match location (*p* < 0.05).

According to the analysis results, more matches were won than drawn or lost 14, 7 and 8, respectively. The team won significantly more matches against high and medium opponent levels (see Figure 2A) and at home than away (see Figure 2B).

**Figure 3:**
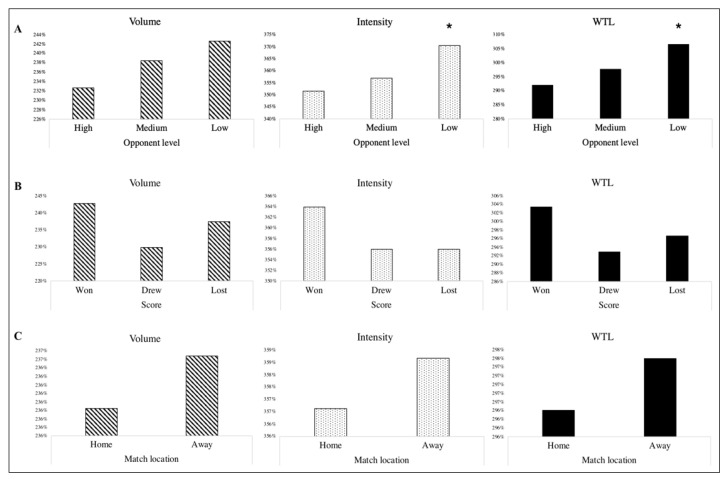
Volume, intensity and WTL according to (**A**) opposition’s level, (**B**) final score and (**C**) match location. * Significant differences between low and high opponent level according to the intensity and WTL (*p* < 0.05).

The intensity of the team was significantly higher in the weeks when the team played against low-level opponents (see Figure 3A). Volume and intensity of the weeks when the team won was higher than when the team lost, although no significant differences were found (see Figure 3B). The microcycles when the team played at home recorded lower levels of volume, intensity and WTL (see Figure 3C), but no significant differences were found.

## 4. Discussion

TL monitoring and assessment of elite players in professional soccer through the use of GPS is well documented and performed on a daily basis [8]. Competitive performance analysis is used as a reference to apply training load in professional soccer players. However, contextual variables condition competitive demands [6,14,15,16,17]. According to it, no study has revealed the cumulative weekly training load of the professional soccer players during the training weeks according to these contextual variables. Retrospectively, in this study, the WTL was analyzed according to the type of week, which were classified according to the contextual variables in line with previous studies [6,15]. Metrics used in this study to quantify TL were selected due to the impact to display the external load accurately reliably and validly in team sports [11,25] and their relation to internal load [10,11].

The mean individual data assessed were then compared to the average maximum individual competitive match-play metric achieved (CP) and reported as a percentage figure, as used in previous studies [8]. According to Owen et al. [8], this method highlights a potential way of monitoring the training patterns of professional soccer players. According to previous research studies, in this study CP was calculated as the maximum values achieved in competition in the 30 analyzed official games [8]. This finding is considered as the best criterion to represent the maximum competitive level reached, knowing that competitive performance varies according to competitive contextual variables [6,14,15,16,17].

According to the analysis of the data, it is possible to think that TL was conditioned by the technical-tactical requirements of the training methodology [1] due to the differences between volume and intensity metrics in training sessions, as occurred in MD. Competitive success in team sports is based on the analysis of the strengths and weaknesses of the team and the opponent. For this reason, contextual variables are aspects to take into account as performance determinants [6,14,17]. Although there are many published studies that analyze the indicators of success in soccer [6], new research trends are based on analyzing success from a complex and multifactorial perspective, generating new hypotheses [1].

Findings of this study showed that the team accumulated less WTL in the weeks where it played against high and medium level teams, being against these opponents when more matches were won. Castellano et al. [15] observed that teams recorded higher high intensity running when faced with top-level and medium-level teams. Findings found in this study showed that WTL, HSR was less in the weeks when there were clashes with high level opponents, with significant differences in accumulated intensity. According to this, we would think that not only the requirement of the opponent conditions the performance of the team in the competition [14,15], but that WTL can be a variable to take into account so that the players can successfully respond to the demands of high intensity of the competition. Several authors determine that reducing HSR and RHSR during the week can be a good strategy to ensure muscle function [27,28].

The findings of this study showed that 79% of the matches won by the team were as the local team (at home), with significant differences. There has been considerable research into home advantage [29], the results suggesting that teams gain a higher percentage of overall points when playing at home [29,30,31]. This study showed that there were no significant differences in WTL when the team played at home. Literature shows that the teams that play at home run more distance and higher intensity than those who play away [15,16]; this finding could be more related to the emotional variables than to the requirement of the training sessions during that week.

Findings from the study concur with traditional planning or tapering models only using one metric methods in reporting the training outcomes, reporting how TL should vary in order to facilitate optimal physiological adaptation. According to this, TL was significantly reduced two days before the match to unload players the day preceding matches in an attempt to increase player readiness [8,11,23]. Managing WTL is considered an important aspect to ensure adaptation processes that cause performance improvements and a reduction in the incidence of injuries [11,32]. To find a balance between fitness and fatigue level is considered an important purpose to reduce the risk of injury [32].

Due to the impact of the contextual variables on the performance, there may be a need to analyze the team and opponents according to these variables to improve the objectivity of the analysis [16,17]. Existing recommendations suggest that the scouting of upcoming opposition should be carried out under circumstances that are reflective of the conditions under which the future match will occur [16]. However, each game is worth three points. According to the results of the study, to train some weeks harder than others may not be a good strategy in a regular competition, because it could cause excessive fatigue to decrement performance.

Considering that competitive performance responses are the main source of information for the planning of training programs [1,6], and that contextual variables modify such responses [15,16,17], it is hoped that the present findings will serve to broaden the body of research on TL/WTL and their possible influence as regard competitive responses according to contextual variables. As such, the results could be used to reduce undesirable effects (for example, by preventing a decline in players’ performance or avoiding an increased likelihood of injury) or to develop management load strategies that help players to maintain their performance in official games.

## 5. Conclusions

The findings of this study showed that competitive performance analysis can be used as a reference to apply training load in professional soccer players.

No previous study had revealed the cumulative weekly training load of the professional soccer players during the training weeks according to the contextual variables. Thus, the findings in this study showed that analyzing success from a complex and multifactorial perspective can generate new hypotheses for training strategies in professional soccer.

According to the results of the study, to train some weeks harder than others may not be a good strategy in a regular competition, because it could cause excessive fatigue to decrement performance.

## Figures and Tables

**Table 1:** Volume and intensity metrics definitions.

	Code	Metrics	Definition
Volume	TDC	Total distance covered (m)	Total distance covered in meters
	ACC	Number of acceleration (m/s^2^)	Number of events >2.50 m/s^2^
	DEC	Number of deceleration (m/s^2^)	Number of events <−2.50 m/s^2^
	HSR	High-speed running (m)	Total distance covered >21 km/h
Intensity	EDI	Equivalent distance index (%)	Ratio between ED (represents the distance that the athlete would have covered at constant speed by using the total energy consumed during a training session) and the TDC
	MP	Metabolic power (W/Kg)	Energy expenditure per unit of time, above resting (speed · energy cost)
	RTDC	Relative distance covered (m/min)	Total distance covered in meters/time
	RHSR	Relative high-speed running (m/min)	Total distance covered >21 km/h/time

**Table 2:** Basic descriptors (mean and standard deviation) for each variable according to the day before match.

	Volume				Intensity			
	TDC ^1^ (m)	Acc (events)	Dec (events)	HSR (m)	EDI	MP (W/Kg)	RTDC (m/min)	RHSR (m/min)
**MD-4**	5260.8 ± 635.7	69.7 ± 14.1	72.6 ± 11.3	174 ± 83	15.1 ± 1	5.7 ± 0.7	78.1 ± 8.4	2.9 ± 1.5
**MD-3**	5889.5 ± 587.8	51.8 ± 8.8	56.9 ± 8.3	313.8 ± 58.3	14 ± 0.9	7.8 ± 0.6	93.2 ± 12.1	4.9 ± 1.1
**MD-2**	3125.2 ± 592.8	28.6 ± 6	30.6 ± 6	98.5 ± 67.5	11.8 ± 1.3	5.1 ± 1	68.1 ± 13.8	2 ± 1
**MD-1**	3725.2 ± 475.1	36.5 ± 3.8	38.1 ± 4.6	182.6 ± 48.3	13.6 ± 48.3	5.3 ± 0.7	73 ± 7.9	3.6 ± 1.1
**MD**	9540.18 ± 201.1	87.6 ± 11.6	100 ± 10.1	496.2 ± 63.4	15.4 ± 1.2	7.7 ± 0.4	101.2 ± 2.2	5.3 ± 0.66

^1^ TDC: total distance covered (m); Acc: total number of high accelerations (>2.50 m/s^2^); Dec: total number of high decelerations (<−2.50 m/s^2^); HSR: total distance covered (>21 km/h); EDI: equivalent distance index; MP: metabolic power; RTDC: relative distance covered; RHSR: relative high-speed running; MD 1–4: match day.

**Table 3:** Volume and intensity markers represented as a % according to the profile competitive.

	Volume				Intensity			
	TDC ^1^	Acc	Dec	HSR	EDI	MP	RTDC	RHSR
**MD-4**	52.7 ± 0.7	58.5 ±1.6	58.8 ± 1.1	27.6 ± 1.7	80.3 ± 0.8	68.7 ± 0.9	75.1 ± 0.8	43.7 ± 2.5
**MD-3**	60.1 ± 0.5	43.5 ± 1.0	46.1 ± 0.8	49.7 ± 1.2	74.6 ± 0.6	92.9 ± 0.8	89.7 ± 1.2	74.6 ± 2.7
**MD-2**	31.3 ± 0.6	24 ± 0.7	24.8 ± 0.6	15.6 ± 1.4	62.9 ± 0.9	61.2 ± 1.8	65.5 ± 1.3	30.5 ± 2.4
**MD-1**	37.4 ± 0.5	30.6 ± 0.4	30.8 ± 0.5	28.9 ± 1.1	72.7 ± 0.8	63.9 ± 0.9	70.3 ± 0.8	55.2 ± 2.5
**MD**	95.7 ± 2.4	73.5 ± 2.7	80.9 ± 2.6	79.0 ± 3.1	84.5 ± 0.7	92.1 ± 0.6	97.4 ± 2.1	80.4 ± 1.3
**WTL**	277.1 ± 1.2	230.2 ± 0.9	241.3 ± 1.8	200.8 ± 1.3	375 ± 0.5	378.8 ± 0.8	398 ± 1.3	284.5 ± 2.1

^1^ TDC: total distance covered (m); Acc: total number of high accelerations (>2.50 m/s^2^); Dec: total number of high decelerations (<−2.50 m/s^2^); HSR: total distance covered (>21 km/h); EDI: equivalent distance index; MP: metabolic power; RTDC: relative distance covered; RHSR: relative high-speed running; MD 1–4: match day; WTL: weekly training load according to the profile competitive represented as a %.

**Table 4:** Description of the WTL in each of the performance variables according to the opponent’s level, final score and location of the match.

		Volume				Intensity				WTL
		^1^ WTL_TDC_	WTL_Acc_	WTL_Dec_	WTL_HSR_	WTL_EDI_	WTL_MP_	WTL_RTDC_	WTL_RHSR_	
Opponent’s level	High	274.5 ± 1.1	227.8 ± 1.3	238.2 ± 1.3	190.3 ± 1.8	371.8 ± 1.3	371.3 ± 1.1	391.1 ± 2.0	271.6 ± 0.3	292.1 ± 0.1
	Medium	280.8 ± 1.1	228.0 ± 1.9	239.6 ± 1.9	205.5 ± 2.2	367.0 ± 1.5	377.7 ± 1.4	397.0 ± 2.0	285.9 ± 0.3	297.7 ± 1.1
	Low	276.4 ± 1.9	236.4 ± 1.5	248.2 ± 1.5	209.6 ± 2.4	381.6 ± 1.1	390.0 ± 1.1	409.6 ± 2.5	300.8 ± 0.3	306.7 ± 1.1 ^$^
Final score	Won	279.9 ± 1.0	236.6 ± 1.4	247.1 ± 1.4	207.7 ± 2.1	376.3 ± 1.3	380.7 ± 1.9	405.3 ± 1.4	293.3 ± 2.5	303.4 ± 0.1
	Drew	269.2 ± 0.4	222.4 ± 1.5	228.1 ± 1.2	199.8 ± 2.6	372.1 ± 1.5	384.3 ± 2.7	385.2 ± 2.6	282.4 ± 4.8	293.0 ± 1.4
	Lost	279.3 ± 1.9	229.9 ± 1.6	244.2 ± 1.6 *	196.2 ± 2.1	370.9 ± 1.3	374.0 ± 1.7	399.8 ± 2.6	279.1 ± 2.9	296.7 ± 1.4
Match location	Home	276.1 ± 0.1	226.7 ± 1.8	239.3 ± 1.6	202.7 ± 2.1	368.2 ± 1.4	379.8 ± 1.8	393.4 ± 1.7	285.1 ± 2.7	296.4 ± 1.0
	Away	277.6 ± 1.7	231.0 ± 1.2	240.9 ± 1.6	196.5 ± 2.4	376.5 ± 1.3	378.9 ± 2.2	399.8 ± 2.5	279.5 ± 3.7	297.6 ± 1.4

^1^ WTL: weekly training load; TDC: total distance covered (m); Acc: total number of high accelerations (>2.50 m/s^2^); Dec: total number of high decelerations (<−2.50 m/s^2^); HSR: total distance covered (>21 km/h); EDI: equivalent distance index; MP: metabolic power; RTDC: relative distance covered; RHSR: relative high-speed running; MD 1–4: match day. * Significant differences between to lose and to draw (*p* < 0.05). ^$^ Significant differences between low and high opponent level (*p* < 0.05).

## Data Availability

The datasets generated and analyzed for this study can be requested by correspondence authors at epardos@usj.es and dlozano@usj.es.
